# Commentary: Concerns regarding the predictive performance of the composite index for mitral regurgitation improvement after TAVI

**DOI:** 10.3389/fcvm.2026.1755552

**Published:** 2026-05-12

**Authors:** Karim Hnid

**Affiliations:** Emergency Department, Moroccan Ministry of Health, Agadir, Morocco

**Keywords:** mitral regurgitation, overfitting, predictive modeling, transcatheter aortic valve replacement (TAVI), TRIPOD guidelines

To the Editor,

I read with great interest the article by Xiao et al., “A composite index for predicting improvement of mitral regurgitation in patients with multivalvular heart disease after transcatheter aortic valve replacement” (Front Cardiovasc Med. 2025;12:1679115). The authors present an index combining persistent atrial fibrillation, eccentric mitral regurgitation, and interventricular septal thickness to predict improvement after TAVI. While the topic is clinically relevant, several methodological issues limit the reliability and generalizability of their findings.

First, the development of a multivariable predictive model using only 53 patients creates a substantial risk of overfitting. According to TRIPOD recommendations, prognostic models should meet adequate sample-size requirements, with at least 10–20 events per variable to ensure model stability (1). In this study, the improvement event occurred in about 36 patients, which is insufficient for a multi-parameter model and strongly predisposes to optimistic estimates.

Second, the authors report an AUC of 0.909 and characterize it as “outstanding discriminative ability”. However, this value was calculated on the same dataset used for model derivation. Without internal validation techniques—such as bootstrap resampling or k-fold cross-validation—the performance is likely optimistic (2). The lack of external validation further limits confidence in the generalizability of the proposed index.

Third, the article implies potential clinical utility of the index. Yet predictive models intended for clinical decision-making must undergo rigorous validation, include calibration assessment, and be tested in independent populations (3). Without these steps, the index cannot be considered ready for clinical application and should instead be viewed as preliminary and hypothesis-generating.

Fourth, qualitative echocardiographic variables such as “eccentric MR” require strict standardization. Echocardiographic assessment of regurgitation is highly dependent on hemodynamic conditions and imaging windows, and inconsistent definitions may introduce classification bias (4).

Finally, the manuscript would benefit from stronger adherence to TRIPOD reporting standards, including transparent reporting of model development, handling of missing data, and justification of sample size (1).

In conclusion, while Xiao et al. address an important question, the composite index should be interpreted with caution due to methodological limitations intrinsic to small-sample predictive modeling without validation. Larger multicenter studies are needed before clinical implementation can be considered.
